# Differential side effects profile in patients with mCRPC treated with abiraterone or enzalutamide: a meta-analysis of randomized controlled trials

**DOI:** 10.18632/oncotarget.20028

**Published:** 2017-08-08

**Authors:** Raphael B. Moreira, Marcio Debiasi, Edoardo Francini, Pier V. Nuzzo, Guillermo De Velasco, Fernando C. Maluf, Andre P. Fay, Joaquim Bellmunt, Toni K. Choueiri, Fabio A. Schutz

**Affiliations:** ^1^ Dana Farber Cancer Institute, Harvard Medical School, Boston, MA, USA; ^2^ Latin American Cooperative Oncology Group, Partenon, Porto Alegre, Brazil; ^3^ Centro Oncológico Antônio Ermírio de Moraes, Beneficencia Portuguesa de Sao Paulo, Sao Paulo, SP, Brazil; ^4^ PUCRS School of Medicine, Porto Alegre, RS, Brazil; ^5^ Hospital Alemão Oswaldo Cruz/Grupo Oncoclinicas do Brasil, São Paulo, SP, Brazil; ^6^ University of Genoa, Genoa, Italy

**Keywords:** metastatic prostate cancer, abiraterone, enzalutamide, toxicity, meta-analysis

## Abstract

**Background:**

Abiraterone and enzalutamide are currently approved for mCRPC patients. Both drugs have distinct mechanisms of action and may have different toxicity profile. There are limited data comparing the side effects of abiraterone and enzalutamide. We performed a meta-analysis of randomized controlled trials (RCT) to better characterize the risk of adverse events associated with both drugs.

**Methods:**

We performed a literature search on MEDLINE for studies reporting abiraterone and enzalutamide side effects from January 1966 to July 31, 2015. Abstracts presented at ASCO meetings from 2004 to 2015 were selected manually. Phase III RCT were included in analysis. We assessed the risk of adverse events reported in RCT by performing two meta-analyses: abiraterone-prednisone vs. placebo-prednisone (2,283 pts) and enzalutamide vs. placebo (2,914 pts). Summary of incidence, relative-risks (RR), and 95% confidence intervals (CI) were calculated using random-effects or fixed-effects models based on the heterogeneity of included studies.

**Results:**

Overall, enzalutamide was not associated with all-grade (RR 1.06 - 95% CI 0.67-1.65) or grade ≥3 (RR 0.81 - 95% CI 0.28-2.33) cardiovascular events, but was associated with increased risk of all-grade fatigue (RR 1.29 - 95% CI 1.15-1.44). On the other hand, abiraterone was associated with increased risk of all-grade (RR 1.28 - 95% CI 1.06-1.55) and grade ≥3 (RR 1.76 - 95% CI 1.12-2.75) cardiovascular events, but was not associated with all-grade (RR 0.85 - 95% CI 0.58-1.23) or grade ≥3 (RR 1.07 - 95% CI 0.97-1.19) fatigue.

**Conclusions:**

In this meta-analysis, abiraterone was associated with an increased risk of cardiovascular events, while enzalutamide was associated with an increased risk of fatigue.

## INTRODUCTION

Prostate cancer is the second leading cause of cancer death among men [1]. Androgen deprivation therapy (ADT) is the standard treatment for advanced prostate cancer. Despite the excellent initial response to ADT, all patients will eventually develop resistance, thus leading to the phenotype of metastatic castration resistant prostate cancer (mCRPC). However, in the last few years, agents targeting the androgen receptor (AR) axis have shown to improve survival of patients with mCRPC [2] [3] [4] [5] [6]. Enzalutamide is a potent direct inhibitor of the AR, while abiraterone is a CYP17A1 inhibitor which in turn decreases androgen synthesis [7] [8]. Both are standard treatments for asymptomatic or mildly symptomatic docetaxel-naive mCRPC, and also for docetaxel treated patients.

Overall, abiraterone and enzalutamide have a favorable toxicity profile. The most frequent adverse events are hot flashes and fatigue for enzalutamide and fluid retention, transaminase increases and hypokalaemia for abiraterone. This topic is of particular relevance in patients with mCRPC, who may have a high number of comorbidities.

In this report, using a meta-analysis design, we sought to investigate the differential incidence of cardiovascular events and fatigue as side effects of abiraterone and enzalutamide in mCRPC patients. The two most important clinical toxicities reported for these drugs are cardiotoxicity for abiraterone and fatigue for enzalutamide. Other exploratory toxicities were not included to avoid the risk of positive findings due to multiple comparisons.

## RESULTS

### Literature search and trials

**Figure 1:**
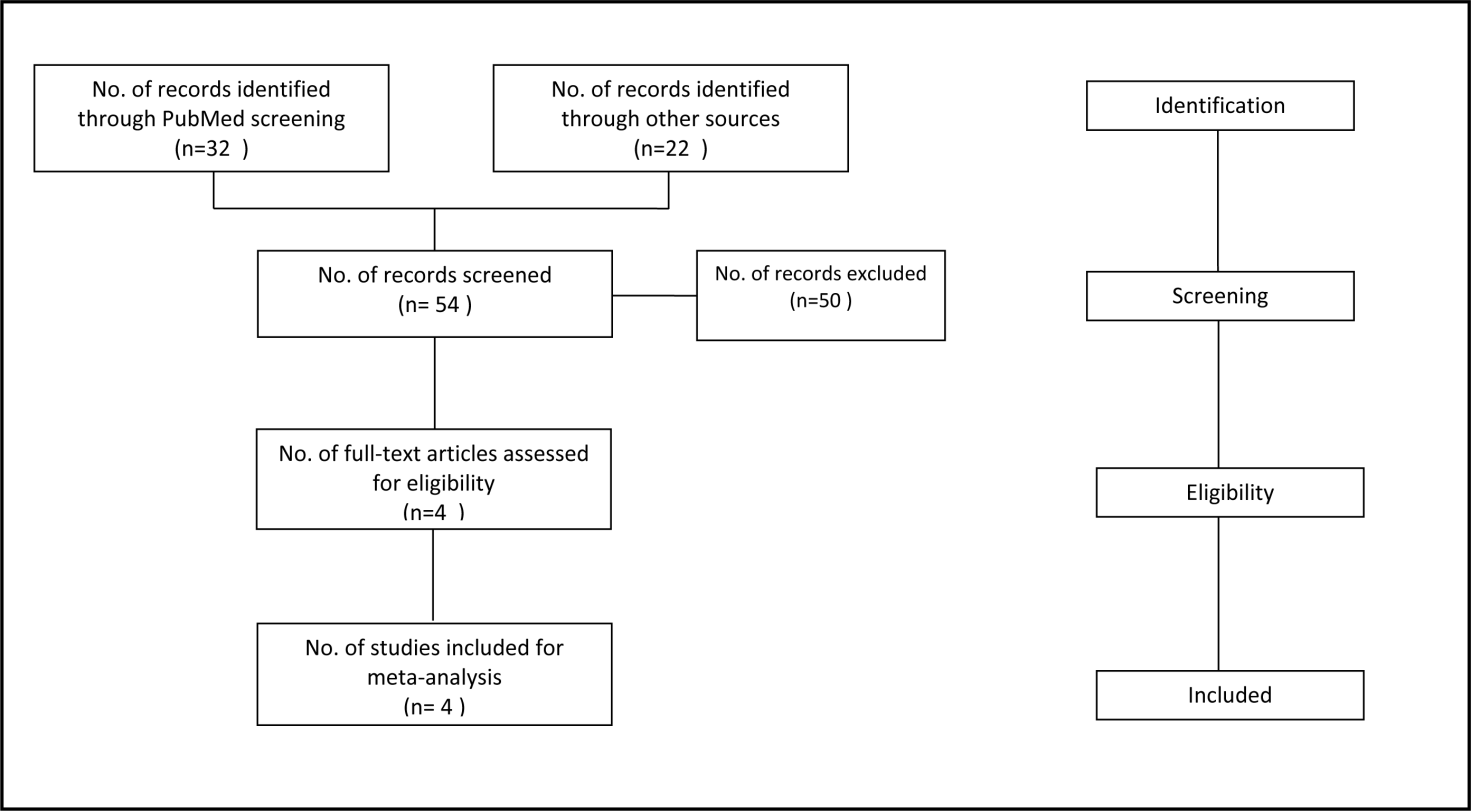
Selection process for randomized controlled trials included in the meta-analysis

Figure 1 describes the flow chart for study selection and Table 1 summarizes the included studies.

**Table 1:** Characteristic of the included studies

Trial	Year	Patients enrolled	Treatment arms (N)	Median age, years (range)	Median PFS (mos)	Median OS (mos)	Median follow-up (mos)	Jadad Score
COU-AA-301 (phase III)	2011	1185	Abiraterone + Prednisone (791)	69 (42-95)	5.6	14.8	12.8	5
			Prednisone (394)	69 (39-90)	3.6	10.9		
COU-AA-302 (phase III)	2013	1082	Abiraterone + Prednisone (542)	71 (44-95)	16.5	nr	22.2	5
			Prednisone (540)	70 (44-90)	8.3	27.2		
AFFIRM (phase III)	2012	1199	Enzalutamide (800)	69 (41-92)	8.3	18.4	14.4	5
			Placebo (399)	69 (49-89)	3.0	13.6		
PREVAIL (phase III)	2014	1717	Enzalutamide (872)	72 (43-93)	11.2	32.4	22.0	5
			Placebo (845)	71 (42-93)	2.8	30.2		

### Abiraterone toxicities

**Figure 2:**
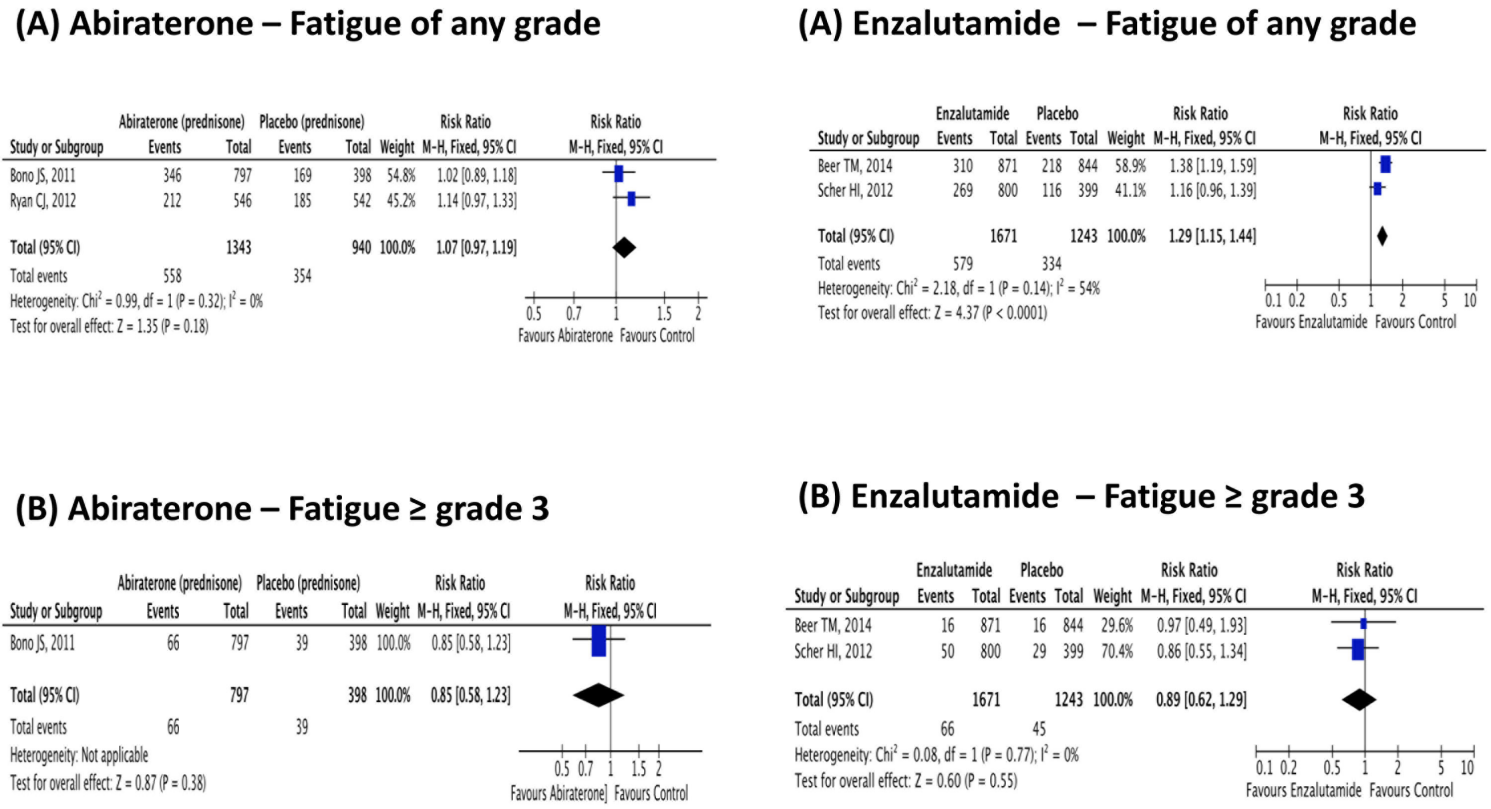
Relative risk for (A) Any grade and (B) ≥ 3 fatigue in patients treated with Abiraterone or Enzalutamide

**Figure 3:**
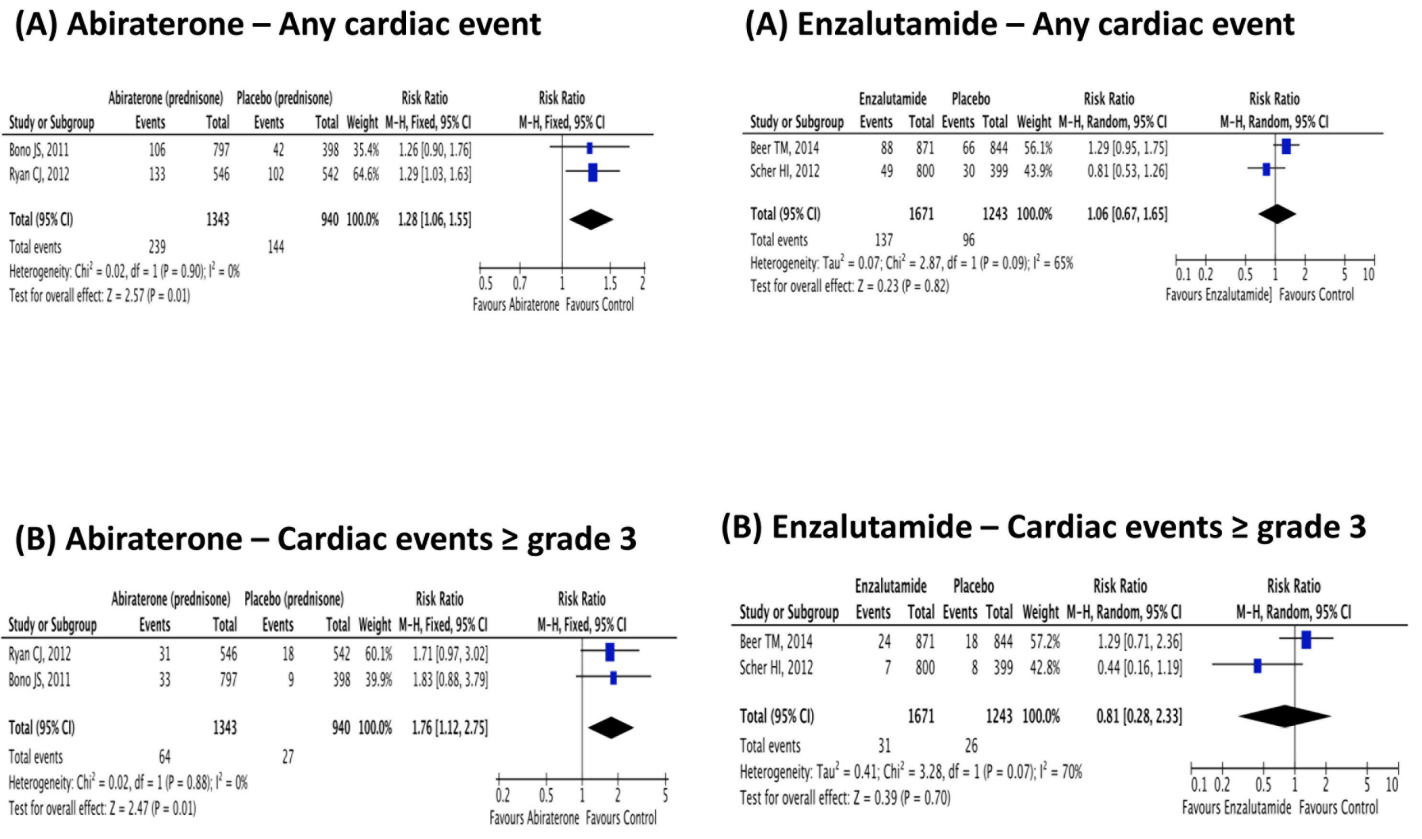
Relative risk for (A) Any grade and (B) ≥ 3 cardiac event in patients treated with Abiraterone or Enzalutamide

Abiratrone was used in association with prednisone and compared to placebo plus prednisone. Abiraterone plus prednisone was associated with a statistically significant 76% increase in the risk of high-grade cardiac disorder adverse events (RR 1.76; IC95% 1.12-2.75; *p* = 0.01) and in a 28% percent increase in all-grade cardiac disorder adverse event (RR 1.28; IC95% 1.06-1.55; *p* = 0.01). On the other hand, abiraterone plus prednisone was neither associated with an increased risk of all-grade nor high-grade fatigue. These results are presented in Figures 2-3.

### Enzalutamide toxicities

Enzalutamide was not associated with an increase in the risk of all-grade or high-grade cardiac disorder adverse event. However, it was associated with an increased risk of all-grade fatigue by 29% (RR 1.29; IC95% 1.16-1.44; *p* < 0.01), but not the risk of high-grade fatigue (RR 0.89; IC95% 0.62-1.29; *p* = 0.55). Figures 2-3 summarizes these results.

## DISCUSSION

To the best of our knowledge, this is the first study to separately evaluate the risk of adverse events reported in RCT of Abiraterone and Enzalutamide. A network meta-analysis was not performed due to the lack of a common arm between trials; Abiraterone plus prednisone was compared to placebo plus prednisone while enzalutamide was compared to placebo alone. The presence of prednisone in one of the placebo arms represents a systematic difference between comparators that would probably interfere with the probability of adverse events in these arms making them impossible to combine. Therefore, we carried out two separate pairwise meta-analyses which demonstrate that patients receiving enzalutamide have a higher risk of all-grade fatigue, but not of all-grade or high-grade cardiovascular events, whereas abiraterone is associated with all-grade and high-grade cardiovascular toxicity, but not with all-grade or high-grade fatigue. With regard to abiraterone cardiovascular side-effects, the incidence and relative risk of cardiovascular events of the new hormonal agents for mCRPC, including abiraterone, have been investigated in a recent network meta-analysis [9]. This study evaluated the toxicities reported in RCT (*n* = 6) of these drugs and reported that their use is associated with an increased risk of all-grade cardiotoxicity (RR=1.32; 95% CI=1.08-1.60; *p* = 0.006) but not with an increased risk of grade ≥3 cardiac events, compared with a placebo. However, the analyzed agents included the novel CYP-17 inhibitor TAK 700 which, due to the negative results in terms of OS achieved, is not approved for the treatment of prostate cancer [10] [11] and, notably, enzalutamide. As mentioned above, the control arms of Enzalutamide RCT differed from those of abiraterone and TAK 700 as it did not include prednisone, which represents a considerable bias for a network meta-analysis as the one performed. At any rate, a sub-analysis of enzalutamide toxicity only from this study observed no significant increase of all-grade or high-grade adverse cardiac events, as proven also in our study [9]. Furthermore, a contemporary metanalysis analyzed the toxicity data from 4 RCT of the CYP-17 inhibitors Abiraterone and TAK 700 in order to determine the incidence and relative risks of side-effects of special interest in patients with mCRPC treated with these agents [12]. Administration of CYP-17 inhibitors was found to be associated with a significant increased risk of all-grade (RR=1.47; 95% CI=1.27–1.7; *p* < 0.00001) and grade ≥3 (RR=1.55; 95% CI=1.18–2.05; *p* = 0.002) cardiovascular disorders. Particularly, in the subset analysis of abiraterone versus TAK 700, a significantly lesser risk of grade ≥3 cardiac adverse events was shown in favor of TAK 700. Taken together, these data seem to support our results. In contrast, in a retrospective study of mCRPC patients with preexisting cardiovascular risk factors treated with abiraterone, no grade ≥3 cardiac toxicity was described. The authors concluded that abiraterone appears a safe option even for patients with cardiac comorbidities [13]. However, the multiple drawbacks of this study, including the small population (*n* = 51), the retrospective analysis, and the fact that all patients cardiac comorbidities were controlled with appropriate therapy prior to starting abiraterone, should be taken into account. Conversely, as the stringent inclusion criteria of RCT do not allow recruitment of patients with inadequate organ function or chronic or concomitant diseases, we deem that our analysis might underestimate the risk of cardiovascular toxicity for the real-world clinical population treated with abiraterone. Furthermore, it should be considered that the vast majority of these patients has been previously exposed to long-term androgen deprivation therapy (ADT) which in several studies was associated with an increased risk of cardiovascular disease [14] [15].

Therefore, we suggest careful consideration of the risks and possible benefits of abiraterone therapy for mCRPC patients with cardiovascular comorbidities (i.e. cardiac ischemia, congestive heart failure, recent myocardial infarction, arrhythmias) or a history of cardiac disease. Lastly, it may be reasonable to recommend performing a detailed cardiovascular assessment prior to starting Abiraterone and periodical monitoring during treatment.

As much as abiraterone-related cardiotoxicity can represent a life-threatening side effect, enzalutamide-related fatigue should not be disregarded. Even if this analysis does not show a higher risk of grade ≥3 fatigue for patients treated with Enzalutamide, our results demonstrate an association with increased risk of all-grade fatigue. It is commonly acknowledged that fatigue may have a major impact on patients’ quality of life [16], self-care abilities [17], and psychological status. That holds particularly true for CRPC patients who are typically elderly, with comorbidities and at an advanced stage of disease. Additionally, most men candidate to enzalutamide have been previously treated with long-term ADT. Fatigue is a common and frequently under-recognized adverse event of ADT [18] and has been consistently described in the literature as enhancing in terms of severity, interference, and duration over the course of ADT [19] [20]. Furthermore, it has been recently proven that a greater number of baseline comorbidities and a higher baseline Gleason score correlate with a worsened fatigue trajectory in men receiving ADT [19]. Therefore, we deem that these risk factors should be part of an accurate screening aimed to select those patients who are less likely to be excessively burdened by this debilitating symptom receiving Enzalutamide. Lastly, fatigue should be adequately monitored by means of validated questionnaires (Cancer Fatigue Scale) throughout treatment.

As both abiraterone and enzalutamide demonstrated similar efficacy prior to and after chemotherapy in mCRPC patients [3] [4], the treatment decision-making should be aided by a careful analysis of patient competing risks of death, chiefly a history of cardiovascular disease or cardiac comorbidities, and patient baseline conditions, particularly fatigue. Besides, a thorough discussion with the patient surrounding the possible risks and benefits of each therapy is warranted and appropriate monitoring during treatment is required.

We are aware that several limitations apply to this study. First, our analysis hinges on solely 4 RCT and not on individual patient data; second, the analyzed RCT adopted different versions of the Common Terminology Criteria for Adverse Events (CTCAE); third, our findings are based on RCT data which might not reflect the real-world setting due to the RCT strict inclusion/exclusion criteria; fourth, other exploratory toxicities were not included.

Despite these drawbacks, our analysis shows an increased risk of all-grade and grade ≥3 cardiovascular events for abiraterone and a higher risk of all-grade fatigue for enzalutamide. These findings provide a better understanding of the divergent side effect profile of these drugs and may help selecting the more appropriate therapy for each patient on the basis of his baseline comorbidities, cardiovascular history, and conditions.

## MATERIALS AND METHODS

### Literature search and study selection

MEDLINE was searched from January 1966 to 31 May, 2016 and ASCO meetings abstracts were manually selected from 2004 to 2016. The MeSH terms used for searching PubMed and the Cochrane Library were ‘abiraterone’ or ‘enzalutamide’. The ASCO University abstracts were searched using the name of the drugs and the terms ‘phase II’ or ‘phase III’. Search was restricted to randomized phase II or III clinical trials reported in English. Title and abstracts of all the original reports identified were reviewed by two independent authors (R.B.M. and F.A.S) for inclusion in this meta-analysis. Cases of disagreement were adjudicated by consensus among reviewers. The full texts of all potentially relevant studies were downloaded and reviwed by two independent authors (R.B.M. and F.A.S) for inclusion in this meta-analysis. Cases of disagreement were adjudicated by consensus among reviewers.

The inclusion criteria for this meta-analysis were: [1] prospective randomized phase II or III trials involving patients with mCRPC and [2] random assignment of patients to study drug (biraterone or enzalutamide) or control. Study quality was assessed by using the seven-point Jadad ranking system that included randomization, double-blinding, and withdrawals, a practice in agreement with other meta-analyses done in this context [21].

### Data extraction

Identified abstracts were then collected and coded by two investigators (R.B.M. and F.A.S) according to the Preferred Reporting Items for Systematic Reviews and Meta-Analyses (PRISMA) statement [22] and any discrepancies between reviewers were resolved by consensus. For each study, the following information was extracted: first author's name, year of publication, trial phase, number of enrolled subjects, number of patients included in the safety analysis, treatment arms, number of patients in abiraterone or enzalutamide treatment and control groups, underlying malignancy, median age, median progression-free survival, overall survival, adverse events of interest.

### Statistical analysis

The outcomes included in this meta-analysis of treatment toxicities were all grade and high-grade any cardiac event and fatigue. Cardiac toxicity was defined by the following events: ischaemic heart disease, myocardial infarction, supraventricular tachyarrhythmias, ventricular tachyarrhythmias, cardiac failure, and possible arrhythmia-related investigations, signs, and symptoms. Treatment effects were pooled as relative risks (RR). All comparisons were based on two-tailed tests and p-values lower than 0.05 were considered significant.

Heterogeneity was accessed using Cochrane Q test and I^2^. Heterogeneity was considered significant whenever the Cochrane Q test yields a p-value under 0.10 or the I^2^ resulted higher than 50%. If heterogeneity was deemed not to be significant, the generic inverse variance fixed effect (Mantel Haenszel) was used; while the use of the random-effect method (DerSimonian) was reserved for cases of significant heterogeneity. Statistical analysis were carried out using the version 5.3 of the software RevMan.
